# Preliminary Evidences of Safety and Efficacy of Flavonoids- and Omega 3-Based Compound for Muscular Dystrophies Treatment: A Randomized Double-Blind Placebo Controlled Pilot Clinical Trial

**DOI:** 10.3389/fneur.2019.00755

**Published:** 2019-07-23

**Authors:** Clementina Sitzia, Mirella Meregalli, Marzia Belicchi, Andrea Farini, Maddalena Arosio, Denise Bestetti, Chiara Villa, Luca Valenti, Paolo Brambilla, Yvan Torrente

**Affiliations:** ^1^Stem Cell Laboratory, Unit of Neurology, Department of Pathophysiology and Transplantation, Centro Dino Ferrari, Università degli Studi di Milano, Fondazione IRCCS Ca' Granda Ospedale Maggiore Policlinico, Milan, Italy; ^2^Service of Physiotherapy, San Raffaele Scientific Institute, Milan, Italy; ^3^Bianchi Bonomi Haemophilia and Thrombosis Center, Fondazione IRCCS Ca' Granda Ospedale Maggiore Policlinico, Milan, Italy; ^4^Department of Pathophysiology and Transplantation, Department of Transfusion Medicine and Hepatology, Translational Medicine, Università degli Studi di Milano, Fondazione IRCCS Ca' Granda, Milan, Italy; ^5^Department of Laboratory Medicine, Desio Hospital, University Milano Bicocca, Milan, Italy

**Keywords:** nutraceutical supplementation, Duchenne muscular dystrophy, safety, tolerability, strength recovery

## Abstract

**Background:** Nutritional compounds can exert both anti-inflammatory and anti-oxidant effects. Since these events exacerbate the pathophysiology of muscular dystrophies, we investigated nutraceutical supplementation as an adjuvant therapy in dystrophic patients, to low costs and easy route of administration. Moreover, this treatment could represent an alternative therapeutic strategy for dystrophic patients who do not respond to corticosteroid treatment.

**Objective:** A 24 weeks randomized double-blind placebo-controlled clinical study was aimed at evaluating the safety and efficacy of daily oral administration of flavonoids- and omega3-based natural supplement (FLAVOMEGA) in patients affected by muscular dystrophy with recognized muscle inflammation.

**Design:** We screened 60 patients diagnosed for Duchenne (DMD), Facioscapulohumeral (FSHD), and Limb Girdle Muscular Dystrophy (LGMD). Using a computer-generated random allocation sequence, we stratified patients in a 2:1:1 ratio (DMD:FSHD:LGMD) to one of two treatment groups: continuous FLAVOMEGA, continuous placebo. Of 29 patients included, only 24 completed the study: 15 were given FLAVOMEGA, 14 placebo.

**Results:** FLAVOMEGA was well tolerated with no reported adverse events. Significant treatment differences in the change from baseline in 6 min walk distance (6MWD; secondary efficacy endpoint) (*P* = 0.033) and in isokinetic knee extension (*P* = 0.039) (primary efficacy endpoint) were observed in LGMD and FSHD subjects. Serum CK levels (secondary efficacy endpoint) decreased in all FLAVOMEGA treated groups with significant difference in DMD subjects (*P* = 0.039).

**Conclusions:** Although the small number of patients and the wide range of disease severity among patients reduced statistical significance, we obtained an optimal profile of safety and tolerability for the compound, showing valuable data of efficacy in primary and secondary endpoints.

**Trial registration number:** NCT03317171

Retrospectively registered 25/10/2017

## Introduction

Muscular dystrophies (MDs) are a heterogeneous group of disorders that lead to muscular weakness and—sometimes—to premature death: although the causative mutations involve different muscular proteins, they share similar fibrotic substitution of degenerated fibers (1) and other secondary aspects as increased reactive oxygen specie (ROS) formation, mitochondria perturbations and, more importantly, sustained inflammation (2). The variability of the clinical phenotype and disease progression may be only partly attributable to the genotype. Variable degrees of muscle involvement were reported in dystrophic patients sharing the same mutation, suggesting that additional genetic or environmental factors may be playing a role in modulating the phenotype. Among the dystrophic features, the muscle infiltration of immune cell is a hallmark of dystrophies and its severity varies among genotypes, even among allelic variants of the same genotype, and among individual patient's muscles or single biopsy, and over time within individual patients. As an example, the rising of inflammation and the consequent activation of the immune system are evident in Duchenne muscular dystrophy (DMD) (2, 3) and in many forms of Limb Girdle Muscular Dystrophies (LGMD) such as dyspherlynopathies (LGMD-2B) (4) calpainopathy (LGMD-2A), and desminopathy (LGMD-2R) (5). Similar to DMD, invasion of macrophages and CD4+ T cells were commonly observed in LGMD biopsies (6). The active role of inflammatory cues was also demonstrated in the early muscular damage of facioscapulohumeral muscular dystrophy (FSHD) (7, 8). Evidence exists for response to immunomodulating therapies such as corticosteroids in some of the MDs (9–13) but are associated with many side effects (13). Future avenues for research include development of new anti-inflammatory drugs targeting the ROS production and NF-κB pathway (14–17). Importantly, cause-specific treatments are currently being investigated in clinical trials to replace the mutated gene using adeno-associated virus (AAV) vector in LGMD patients (ClinicalTrials.gov identifiers: NCT03492346 and NCT03652259) (18) and to restore the correct open-reading frame of dystrophin pre-mRNA in DMD patients (19, 20). In particular, Eteplirsen (an anti-sense oligonucletide) was recently approved by the Food and Drug Administration (FDA) to treat approximately 14% of patients with DMD mutations that can be restored through specific skipping of exon 51 (21, 22). Similarly, the European Medicines Agency approved the Ataluren, specifically designed to allow readthrough of nonsense mutations in DMD patients (23, 24). However, muscle inflammation represent a hostile and detrimental environment for the efficacy of these new therapies. Chronic inflammation increases the bioenergetics metabolic rate and demand of energy of MD patients (25). Supplements such as Coenzyme Q10 (CoQ10), carnitine, amino acids (glutamine, arginine), anti-inflammatory/antioxidants (fish oil, vitamin E, polyphenols) have been proposed as dietary treatment which affects the level of metabolites, reduces free radicals, or stimulates antioxidant enzymes (26–30). Furthermore, supplementation with curcumin or creatine plus CoQ10 reduces NF–κB activity and modulates mitochondrial respiratory chain function with energy production (31). Interestingly, combinatorial therapies with amino acids plus deflazacort have been proposed in DMD patients to improve nitrogen retention and maintain protein balance (32–35). However, since dosage and safety requirements as stringent as those needed for pharmacological compounds are not necessary in the field of nutritional interventions, clinical investigations of supplement diet effects in MDs are mandatory to determine the most valuable ones. We previously demonstrated that a combination of flavonoids (curcumin, baicalin, and green tea) and omega3 as main compounds ameliorates dystrophic features in the animal model of DMD, the mdx mouse. This treatment improved endurance and muscular features by reducing muscle fiber necrosis and fibrosis deposition and by increasing muscle mass. Although the mechanism of action is not completely clarified, we demonstrated its scavenger activity on ROS production and its anti-inflammatory properties regulating the recruitment of inflammatory cells in muscle tissues (36). In another work, we showed that mdx supplemented with specific branched-chain amino acid-enriched mixture (BCAAem) ameliorated the pathological phenotype (37). Here we report the results of a single center randomized double-blind placebo controlled study planned to validate our preclinical evidences (36) and assess the safety and partially the efficacy of dietary supplementation of flavonoids- and omega3-based natural supplement (hereafter referred to as FLAVOMEGA) in 29 dystrophic patients diagnosed for DMD, FSHD, and LGMD.

## Subjects and Methods

### Trial Registration: Standard Protocol Approvals, Registrations, and Patient Consents

This phase II, randomized double-blind placebo-controlled clinical study was conducted at the Fondazione IRCCS Ca' Granda Ospedale Maggiore Policlinico, Milan, Italy. Each patient (or patient's parents) gave the written informed consent for the research. This study was performed in accordance with International Conference on Harmonisation of Good Clinical Practice guidelines, the Declaration of Helsinki (2008) and the European Directive 2001/20/EC. This monocenter study was approved by the Ethical Committee at Fondazione IRCCS Ca' Granda Ospedale Maggiore Policlinico of Milan, under the acronym PRO1. This trial was registered on ClinicalTrials.gov with the following number: NCT03317171.

### Data Sharing and Data Accessibility

All data generated or analyzed during this study are included in this published article (and its Supplementary Information Files) or available from the corresponding author on reasonable request.

### FLAVOMEGA

UGA Nutraceuticals Srl and Ystem Srl were responsible for the production and release of placebo and dietary supplement. FLAVOMEGA and placebo composition are reported in Tables 1, 2. FLAVOMEGA dose: sachet 1, powdered phase: 80 g net weight per day. Ingredients: fructose, phospholipidic curcumin, Acetil carnitin-l-HCL, ascorbic acid, flavoring, CoenzymeQ10, Skullcap (*Scutellaria baicalensis* Georgi) Baicalin, Green Tea (*Camellia Sinensis*) cathechins, anti-agglomerant: silicon dioxide, edulcorant: acesulfame potassium and sucralose. Sachet 2, oily phase: 81.4 g net weight per day, Ingredients: fish oil 05/25, Vitamin E acetate, citrus (*Citrus Limonum*) essential oil.

**Table 1:** FLAVOMEGA composition.

**Oily phase**	**Ingredient**	**For 100 ml**	**Daily dosage (stick-pack)**
	DHA	19.231 g	1,250 mg
	EPA	6 g	360 mg
	Vitamin E	0.554 g	36 mg
	Lemon essential oil	0.114 g	7.395 mg
	**Total**		6.5 ml
**Powdered phase**	**Ingredient**	**For 100 g**	**Daily dosage (stick-pack)**
	Curcumin complexed with phospholipid	20.000 g	1000.000 mg
	Acetyl L-Carnitine	15.000 g	750.000 mg
	Ascorbic acid	4.800 g	240.000 mg
	Coenzyme Q10	4.000 g	200.000 mg
	Dry extract of the roots of scutellaria	2.106 g	105.300 mg
	Dry extract of green tea	2.000 g	100.000 mg
	**Total**		5 g

*DHA, docosahexaenoic acid; EPA, Eicosapentaenoic acid*.

**Table 2:** Placebo composition.

**Oily phase**	**Ingredient**	**For 100 ml**	**Daily dosage (stick-pack)**
	Sunflower oil (*Helianthus annuus L*.)		6.436 g
	Lemon essential oil		0.064 g
	**Total**		6.5 ml
**Powdered phase**	**Ingredient**	**For 100 g**	**Daily dosage (stick-pack)**
	Fructose	44.600 g	2230.000 mg
	Micro-crystalline cellulose	36.000 g	1800.000 mg
	β-carotene [E160a(i)]	8.000 g	400.000 mg
	Orange flavor	6.000 g	300.000 mg
	Cytric acid	4.000 g	200.000 mg
	Silicon bioxide (E551)	1.000 g	50.000 mg
	Sucralose (E955)	0.400 g	20.000 mg
	**Total**		5 g

### Clinical Trial Designation: the Characteristics of Participants

**Figure 1:**
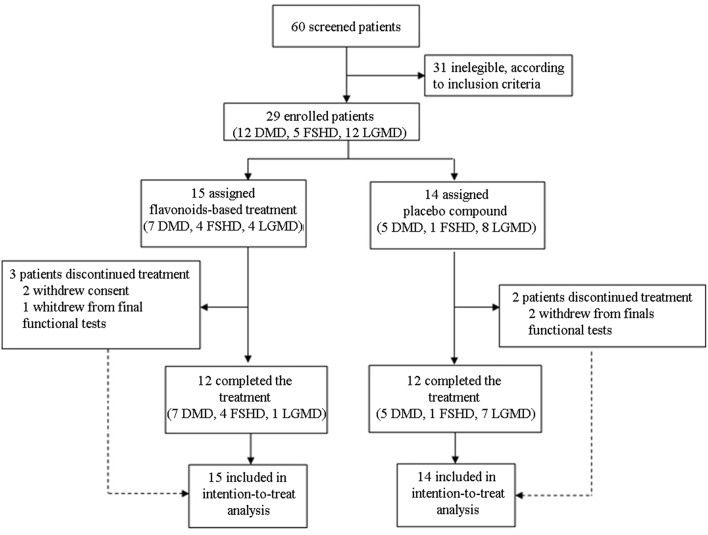
CONSORT flow diagram.

We designed a phase II, randomized double-blind placebo-controlled clinical study to assess the safety and efficacy of one dosing regimen of FLAVOMEGA oral administration in 29 patients affected by DMD, FSHD, and LGMD (Figure 1). We screened 60 DMD, FSHD, and LGMD patients who were characterized at the level of their gene mutations and skeletal muscle biopsy in 6 specialist centers in Italy. Included 29 patients were evaluated at baseline and after 24 weeks of treatment. Inclusion criteria featured documented genetic diagnosis and histological confirmation for inflammatory mononuclear cellular infiltrates in muscle biopsy of DMD, FSHD, and LGMD patients; age superior to 9 years for DMD, between 9 and 70 years for LGMD, and between 20 and 70 years for FSHD; absence of severe cardiac and pulmonary disease. In particular, we defined cardiomyopathy with values of left ventricular ejection fraction (EF) and shortening fraction (SF) of, respectively, <55 or <28% or both, while severe cardiomyopathy was considered with EF <45% and SF <20%. Severe pulmonary disease was determined by Forced Expiratory Volume in 1 s (FEV_1_) <0.8 L and Force Vital Capacity (FVC) <40% (38–40). Included DMD patients' genotyping showed deletion of *N*-terminal (*N* = 3), ROD domain (*N* = 8), and point mutation of ROD domain (*N* = 1) of dystrophin gene. Included LGMD patients' genotypes were LGMD-2B (*N* = 6), LGMD-2A (*N* = 5), and LGMD-2R (*N* = 1). Included FSHD patients presented reduced numbers of repetitive units of D4Z4 allele <10 (*N* = 5). Five out 12 LGMD and all FSHD enrolled patients were males.

Patients treated with corticosteroids for <6 months before study were not included to avoid bias on FLAVOMEGA effects and corticosteroid treatment was maintained for the duration of the study: of screened patients, only four DMD patients were on corticosteroid with a median prednisolone adjusted dose of 11.5 mg/kg/month (range 7.5–24). Steroid dosage was not changed during the study. None of the subjects had health problems other than muscular dystrophy. Vital signs such as oxygen saturation, respiratory frequency, body temperature, and diastolic blood pressure were normal for age. All included DMD patients were non-ambulant and no one had severe scoliosis or severe contractures that could have limited the use the assessment of strength.

Baseline cardiological examination, ECG and echo showed right bundle branch block in 5 subjects, minimal mitral valve insufficiency in 4 subjects, and posterior wall hypertrophy of no clinical relevance in 6 subjects. Pulmonary function tests evaluable for 22 subjects were normal in 17 subjects while 5 subjects had minimal respiratory insufficiency. We obtained written informed consent from each patient's parents or guardians before any study procedure was undertaken; we obtained written consent from patients when appropriate.

Using a computer-generated random allocation sequence, we accordingly stratified patients in a 2:1:1 ratio (block size of six; DMD, FSHD, and LGMD stratification) to one of two treatment groups: continuous FLAVOMEGA, continuous placebo. FLAVOMEGA and placebo oral administration had identical packaging and flavor solutions. Efficacy assessment was performed by personnel not involved in the general clinical and safety assessment of patients. Sponsor personnel who did not have any direct interactions with the investigator site was unmasked to efficacy and safety data after week 24, when the database was first locked. However, masking was maintained at study sites (for patients, their families, investigators, and any personnel with direct contact with the site) until final database lock at week 55. Safety and tolerability endpoints included adverse event (AEs), serious adverse event (SAEs), laboratory parameters, and physical examination.

A number of scales exist to evaluate muscle impairment of MD patients; similarly, different scores are taken in considerations and widely used for neuromuscular diseases, as reported in literature (41–44). However, for the prospective analysis, the functional scores needed to cover the full spectrum of abilities (i.e., upper and lower limbs). According to these evidences and considering the proximal weakness as clinical hallmarks of DMD, FSHD, and LGMD, our clinical efficacy endpoints included the assessment of endurance by 6MWD (6 min walking distance) in walking patients and dynamometer muscle strength measurement of upper and lower limbs in both walking and wheelchair patients. The parent questionnaire data (EK, Egen Klassifikation; ACTIVLIM, Activity Limitation; ABILHAND, manual ability for adults with upper limb impairments) scales were also used as previously described (45–48). During a 24 weeks period, all patients were administered daily with a dose of either treatment or placebo, which they independently took in. Two daily combined sachets, respectively, powdery and oily phase, were provided for each treatment. Sachets of supplement and placebo were visually identical. We performed safety and efficacy assessments at screening baseline and week 24. 6MWD and dynamometer muscle strength measurements of elbow and knee flexo/extension were tested as previously described (49, 50). The 6MWT reflects the physical capacity and walking function at a submaximal level, evaluates the global and integrated responses of all the systems involved during exercise, including the pulmonary and cardiovascular systems and—this way—it was accepted as a clinically meaningful outcome measure by the regulatory authorities (51). For the isometric knee (elbow) extension/flexion, MDs' patients were seated on Biodex chair, leg (arm) were positioned with knee (elbow) at 90° aligned to dynamometer arm and the isometric tetanic voluntary contraction of related extensor and flexor muscles were recorded three times/exercise with 5 s of rest between one cycle and another. For the isokinetic knee extension/flexion measurement, during the isokinetic voluntary contraction of leg extensor or flexor muscles, speed was set and maintained at 20 m/sec. Both upper and lower limbs were analyzed. EK, ACTIVLIM, and ABILHAND scales were submitted to all included patients at baseline and 8, 16, and 24 weeks later as previously described (46). Blood analysis were performed at baseline and 24 weeks later using Sysmex XE-2100 (DASIT), CBC (hemoglobin), and COBAS analyser (Roche Diagnostic) at Lab Analysis unit, Fondazione IRCCS Ca' Granda Ospedale Maggiore Policlinico. Serum levels of β-hydroxybutyrate, free fatty acid (FFA) and branched-chain amino acids (BCAA) were measured by gas chromatography-mass spectrometry using the Agilent GCMS 5973N mass spectrometer system, as described in detail elsewhere (52). Commercially available kit for ROS detection was used (Ros-Glow^TM^ H_2_O_2_, Promega).

### Objectives of the Study

Our primary objectives were to assess the safety and efficacy of FLAVOMEGA supplementation after 24 weeks of treatment. The primary endpoints were to verify safety by any significant changes in blood tests and appearance of side effects and to verify any significant changes in Biodex System 4 Dynamometer functional test from baseline to week 24. The secondary efficacy endpoints were set to verify modifications in 6MWT and CK concentrations. Exploratory efficacy endpoints included the EK, ACTIVLIM, and ABILHAND scales (46). Physical examinations included electrocardiograms (ECGs).

### Statistical Considerations

The study was exploratory and not designed or powered to show a statistically significant difference in the primary and secondary endpoints between treatment groups. For the primary and secondary endpoints analysis, we analyzed the data using linear regression for repeated measures (MMRM) and *t* test analysis with Prism Software and SPSS Software, calculating mean and standard deviation (SD) at baseline in Intended to treat population (ITT) and adjusted mean for missing value at baseline and T24 weeks. For each analysis, we indicated regression coefficient and standard error (SE) and *p*-value. 95% Confident interval (CI) was considered in each test. For creatine kinase, linear regression was applied using absolute difference values (value at T24-value at baseline/value at baseline) as independent variable to normalize data. For 6MWT, linear regression was applied using difference in meters walked as independent variable and treatment and baseline meters walked were meant as dependent variables. Biodex strength analysis was conducted normalizing data on baseline value (unpaired *t*-test) or by pairing patients at baseline and at T24 weeks (paired *t*-test).

## Results

We screened 60 patients and included 29 patients in accordance with the study inclusion criteria, from Sept 7, 2015, until January 10, 2016. The final number of patients completing the study was 24 (Figure 1). At the baseline, the mean (±SD) age for the total study population was 34.72 ± 15.49 years (range 15–67). Mean ± SD age per disease stratification was 20.17 ± 4.78 for DMD (*N* = 12), 36.80 ± 9.83 for FSHD (*N* = 5), and 48.42 ± 10.73 for LGMD (*N* = 12). Demographic characteristics were much the same across DMD treated group, while LGMD group had a greater mean age than the other DMD and FSHD groups, as well as time since first symptoms and diagnosis (Table 3). Gender difference was present only in LGMD group, which was composed of 5 males and 7 females (Table 3). All DMD and 5 LGMD patients were non-ambulant at baseline, while all FSHD patients were ambulant. 4 LGMD patients withdrew from the study at different time points during the 6 months period; 1 FSHD patient did not undergo final functional tests (Figure 1). Patients' features before or after group stratification are summarized in Tables 3, 4. Patients' strength measurement at baseline after stratification are summarized in Tables 5, 6 and Supplementary Table 1. No adverse events were reported. No clinically significant changes from baseline data were observed on physical examination, in vital signs, or ECGs (data not shown). No gender-related differences in these values were found in LGMD patients. No blood test modification was observed and none of the patients showed liver-enzyme changes suggesting hepatotoxicity (Table 7). All these data demonstrated the safety and high-tolerability of FLAVOMEGA in included dystrophic subjects.

**Table 3:** OD patients features at baseline.

**Baseline**	**LGMD**	**FSHD**
Number	12	5
Age (years ± SD)	48.41 ± 10.73	36.8 ± 9.8
Sex	Male 5/12	Male 5/5
Race	Caucasian	Caucasian
Weight (kg ± SD)	71.66 ± 8.95	72.4 ± 15.32
Walking ability	7/12	5/5
Corticosteroids assumption	0/12	0/5
Years since diagnosis (±SD)	22 ± 7.68	10 ± 7.17
Strength measurement (±SD)	19.42 ± 19.91	100.42 ± 54.52

*SD, Standard Deviation*.

**Table 4:** Patients features description at baseline.

**Baseline**	**DMD**		**OD**	
**ITT**	**Placebo**	**FLAVOMEGA**	**Placebo**	**FLAVOMEGA**
Patients (*N*=)	5	7	9	8
Age (years ± SD)	22.6 ± 2.6	18.43 ± 1.25	44.77 ± 10.23	45.25 ± 13.58
*t*-test (*P*-value)	*P* = 0.0039^*^		*P* = 0.935	
Sex	Male	Male	Male 5/9	Male 5/8
Race	Caucasian	Caucasian	Caucasian	Caucasian
Weight (kg ± SD)	59.8 ± 7.46	54.43 ± 8.08	67.22 ± 7.69	77.13 ± 11.54
Walking ability	0/5	0/7	8/9	4/8
Strength measurement (Biodex) (N/m ± SD)^§^	5.57 ± 1.69	5.89 ± 4.03	21.30 ± 24.30	60.43 ± 63.04
*t*-test (*P*-value)	*P* = 0.871		*P* = 0.107	
Corticosteroids assumption	2/5	2/7	0/9	0/8
Year since diagnosis (±SD)	16 ± 3.79	15 ± 2.76	17 ± 6.25	20 ± 11.80
Serum CK (U/L ± SD)^**^	1051.00 ± 693.17	2402.29 ± 1146.79	627.00 ± 185.03	639.36 ± 642.84
*t*-test (*P*-value)	*P* = 0.042^*^		*P* = 0.956	
6MWT (meter walked)^**^	Np	Np	313.57 ± 214.19	568.25 ± 130.76
*t*-test (*P*-value)	Np		0.0617	

* *statistically significant*.

§ *Mean of global analysis*.

** *Per Protocol*.

*ITT, intended to treat population; SD, Standard Deviation*.

**Table 5:** Global strength of all included patients at baseline.

**ITT**	**Placebo**	**FLAVOMEGA**
Patients (*N*=)	14	15
Strength measurement Biodex (N/m ± SEM)	18.12 ± 5.72	33.16 ± 13.74
Difference between means	15.04 ± 14.88	
95% confidence interval	−15.55 to 45.64	
*t*-test (*P*-value)	*p* = 0.321	
**PER PROTOCOL**		
Patients (*N*=)	12	12
Strength measurement Biodex (N/m ± SEM)	14.75 ± 6.18	37.43 ± 14.84
Difference between means	22.68 ± 16.08	
95% confidence interval	−10.66 to 56.02	
*t*-test (*P*-value)	0.172	

*SEM, Standard error of the mean*.

**Table 6:** Global strength of DMD and OD included patients at baseline and the values concerning the single Biodex measurement.

	**DMD**							**OD**						
	**Placebo**			**FLAVOMEGA**				**Placebo**			**FLAVOMEGA**			
**Baseline per protocol analysis**	**Mean**	**SD**	N^**^	**Mean**	**SD**	***N***	***P*-value**	**Mean**	**SD**	***N***	**Mean**	**SD**	***N***	***P*-value**
Global strength measurement Biodex (N/m)	5.57	1.69	5	5.89	4.03	7	0.871	21.3	20.73	7	81.58	63.27	5	0.0381^*^
Isometric knee extension R	5.98	1.11	4	3.78	5.69	7	0.473	39.84	31.26	5	178.61	118.13	5	0.034^*^
Isometric knee extension L	9.86	7.35	3	6.38	3.53	7	0.322	38.99	34.31	7	146.27	131.07	5	0.061
Isometric knee flexion R	8.59	6.86	3	11.36	7.49	7	0.599	15.44	12.90	7	76.28	51.39	5	0.012^*^
Isometric knee flexion L	7.25	5.21	2	9.52	5.58	5	0.643	14.50	16.94	7	78.52	42.62	4	0.005^*^
Isokinetic knee extension R	7.61	3.30	3	9.74	1.93	4	0.326	28.14	27.72	7	104.16	94.21	4	0.069
Isokinetic knee flexion R	9.31	1.63	3	13.22	10.87	4	0.572	19.28	17.42	7	64.67	47.65	4	0.044^*^
Isokinetic knee extension L	4.68	0.87	3	11.37	0.64	4	0.001^*^	34.29	33.24	7	119.02	123.17	5	0.182
Isokinetic knee flexion L	5.70	0.80	3	11.76	5.25	4	0.110	22.17	22.58	7	55.41	52.44	5	0.161
Isometric elbow extension R	2.66	0.66	5	3.90	2.72	7	0.347	15.45	13.45	6	29.15	30.69	5	0.346
Isometric elbow extension L	3.89	3.27	5	3.73	2.77	7	0.928	13.95	13.21	7	25.51	28.06	5	0.357
Isometric elbow flexion R	5.20	/^§^	1	5.28	0.95	4	/^§^	14.20	14.99	7	47.61	31.88	4	0.039^*^
Isometric elbow flexion L	5.15	/^§^	1	2.65	0.07	3	/^§^	13.66	16.52	7	39.24	31.65	4	0.105

* *Statistically significant*.

** *N, number of patients that performed the test (observations)*.

*P-value is intended as unpaired t-test P-value*.

/§ *Values are incalculable due to paucity of observations*.

*SD, Standard Deviation; R, right; L, left*.

**Table 7:** Linear regression of repeated measure (MMRM) analysis of secondary endpoints: safety blood test parameters.

**AST (U/L) (RV: 29 U/L)**	**Placebo**	**FLAVOMEGA**
Patients (*N*=)	12	13
Mean baseline	41 ± 22.65	51.65 ± 28.35
Mean T 24wk (*N*=)	39.44 ± 17.78 (9)	41.16 ± 15.90 (8)
Mean difference	−1.37	−8.51
*P*-value (95% CI)	0.651 (−8.239 to 5.489)	0.2901 (−26.10 to 9.076)
**ALT (U/L) (RV: 36 U/L)**		
Patients (*N*=)	11	14
Mean baseline	57 ± 30.08	68.75 ± 43.34
Mean T 24wk (*N*=)	46.89 ± 22.21 (8)	57.62 ± 23.78 (9)
Mean difference	−6.43	−11.78
*P*-value (95% CI)	0.2378 (−18.43 to 5.569)	0.2781 (−35.12 to 11.56)
**GGT (U/L) (RV: 55 U/L)**		
Patients (*N*=)	14	12
Mean baseline	35.14 ± 27.78	22.675 ± 10.71
Mean T 24wk (*N*=)	33.27 ± 16.15 (11)	23.58 ± 8.80 (9)
Mean difference	−3	1.51
*P*-value (95% CI)	0.6309 (−16.49 to 10.49)	0.4597 (−3.057 to 6.080)
**Creatinin (mg/dL) (RV: 0.35–1.2 mg/dL)**		
Patients (*N*=)	14	14
Mean baseline	0.37 ± 0.25	0.37 ± 0.31
Mean T 24wk (*N*=)	0.40 ± 0.23 (8)	0.38 ± 0.32 (7)
Mean difference	0.02	0.007
*P*-value (95% CI)	0.4015 (−0.025 to 0.055)	0.9226 (−0.165 to 0.179)
**Hgb (g/dl) (RV: 11–18 g/dl)**		
Patients (*N*=)	14	14
Mean baseline	14.45 ± 1.30	14.65 ± 1.53
Mean T 24wk (*N*=)	14.31 ± 1.22 (10)	14.83 ± 1.46 (9)
Mean difference	0.11	0.038
*P*-value (95% CI)	0.6311 (−0.391 to 0.611)	0.8444 (−0.398 to 0.473)
**RED cells (×10**^**6**^**/μl) (RV: 4.5–6** **×10**^**6**^**/μl)**		
Patients (*N*=)	13	14
Mean baseline	5.01 ± 0.47	5.06 ± 0.54
Mean T 24wk (*N*=)	4.90 ± 0.32 (10)	5.06 ± 0.38 (9)
Mean difference	6.26	−2.176
*P*-value (95% CI)	0.3744 (−8.740 to 21.26)	0.1112 (−5.000 to 0.648)
**White cells (×10**^**3**^**/μl) (RV: 4.5–11** **×10**^**3**^**/μl)**		
Patients (*N*=)	14	14
Mean baseline	6.90 ± 1.42	7.60 ± 2.60
Mean T 24wk (*N*=)	6.12 ± 1.20 (7)	7.89 ± 2.36 (8)
Mean difference	−0.36	0.49
*P*-value (95% CI)	0.1787 (−0.927 to 0.216)	0.2436 (−0.584 to 1.559)
**Platelets (×10**^**3**^**/μl) (RV: 150–400** **×10**^**3**^**/μl)**		
Patients (*N*=)	14	14
Mean baseline	250.07 ± 41.50	254.85 ± 64.73
Mean T 24wk (*N*=)	230.09 ± 47.54 (11)	229.22 ± 42.79 (9)
Mean difference	−22.45	−25.25
*P*-value (95% CI)	0.1073 (−50.73 to 5.826)	0.35 (−84.88 to 34.38)

*AST, aspartate aminotransferase; ALT, alanine amino-transferase; GGT, gamma glutamyl transferase; Hgb, hemoglobin; CI, Confidence Interval*.

Efficacy of the treatment was analyzed considering all patients together, without respect to their specific pathology or mutations, as FLAVOMEGA is thought to exert a broadly active effect on inflammation and metabolism. Deeper analysis was conducted separately on DMD group trying to highlight the effectiveness of treatment on patients gathered with similar biomechanical features (i.e., low force and loss of ambulation). Similar analysis was conducted on LGMD and FSHD groups. However, in these latter cases, considering that randomization did not account for patients' mutation and the paucity of subjects, we could not separately evaluate these groups. This way LGMD and FSHD patients were considered together and termed as Other Dystrophies (OD). Nevertheless, both LGMD and FSHD patients present high intra-group variability in terms of age and force although they share similar characteristics (still ambulant, middle-adult age, greater force than DMD group) (Table 3).

Firstly, global strength analysis at T24 weeks (calculated as the average of each patients' outcomes) performed considering all patients belonging to FLAVOMEGA vs. placebo groups demonstrated a statistically significant effect of treatment in increasing muscle performance (Wilcoxon test on paired data, *P* = 0.0396). This data was confirmed in FLAVOMEGA treated DMD group (Wilcoxon test on paired data, *P* = 0.0034) whereas no significant increase of global strength was observed in FLAVOMEGA treated OD group (Wilcoxon test on paired data, *P* = 0.470) (Table 8).

**Table 8:** FLAVOMEGA effect at T24 weeks on global strength analysis.

**Paired all patients mean global strength**	**Placebo**	**FLAVOMEGA**
Mean difference	−1.622	−0.553
*P*-value Wilcoxon Signed rank test		^*^0.0396
*N* = 24		
**Paired OD mean global strength**		
Mean difference	−1.907	−0.448
*P*-value Wilcoxon Signed rank test		0.470
*N* = 12		
**Paired DMD mean global strength**		
Mean difference	−1.226	−0.63
*P*-value Wilcoxon Signed rank test		^*^0.0034
*N* = 12		

* *statistically significant*.

*SEM, Standard error of the mean*.

Similarly, Paired *t*-test analysis of the quantitative dynamometer strength measures (calculated as the average of each exercise) demonstrated a stabilization of muscle force in FLAVOMEGA treated patients while there was a significant decrease in all placebo patients (mean of differences: −1.84 N/m, *P* = 0.002) or DMD placebo patients (mean of differences: −0.89 N/m, *P* = 0.051), and OD placebo patients (mean of differences: −2.23 N/m, *P* = 0.0008) (Table 9 and graph in Supplementary Figure 1).

**Table 9:** Biodex measurement of strength N/m (paired *t*-test) considering all muscular districts before and after treatment (baseline and T24wk) intra each group.

**Strength (N/m) all muscular district**	**Paired baseline vs. T24 weeks**	
**All patients**	**Placebo**	**FLAVOMEGA**
Mean of differences	−1.84	−0.047
SD of differences	5.06	19.64
SEM of differences	0.47	1.839
95% CI	−2.78 to −0.90	−3.69 to 3.60
*n* of pairs	114	114
*P*-value	^***^0.0002	0.9798
**DMD**		
Mean of differences	−0.89	−0.42
SD of differences	2.52	3.76
SEM of differences	0.44	0.49
95% CI	−1.78 to 0.004	−1.39 to 0.55
*n* of pairs	33	60
*P*-value	0.051	0.391
**OD**		
Mean of differences	−2.23	0.37
SD of differences	5.75	28.39
SEM of differences	0.64	3.86
95% CI	−3.50 to −0.96	−7.38 to 8.12
*n* of pairs	81	54
*P*-value	^***^0.0008	0.92

*** *statistically significant. SD, Standard Deviation; SEM, Standard error of the mean; CI, Confidence Interval; OD, other dystrophies (FSHD and LGMD)*.

DMD patients showed similar strength values as they are all non-ambulant and of similar age, while a greater variability was present in OD group as it includes both LGMD and FSHD patients with different degree of severity of the disease (Table 9 and Supplementary Figure 1). Next, we analyzed whether the effect of FLAVOMEGA on strength differed between upper and lower limbs. We evaluated the mean change of force from baseline by analyzing with unpaired *t*-test the ratio of quantitative dynamometer parameter at T24/T-baseline. Although upper-limbs strength remained unchanged during the study, statistically significant differences of quantitative dynamometer strength of lower limbs were found in all treated vs. placebo groups (*P* = 0.047). Moreover, statistically significant differences of quantitative dynamometer strength of lower limbs were also found in OD treated vs. OD placebo group (*P* = 0.038) (Table 10 and graph in Supplementary Figure 2).

**Table 10:** Biodex measurements of strength as T24wk/baseline ratio and unpaired *t*-test between Placebo and FLAVOMEGA treated groups.

**Strength (T24wk/baseline)**	**All muscular district**		**Lower limbs**		**Upper limbs**	
**All patients**	**Placebo**	**FLAVOMEGA**	**Placebo**	**FLAVOMEGA**	**Placebo**	**FLAVOMEGA**
Mean	0.99 ± 0.05 *N* = 113	1.23 ± 0.14 *N* = 112	0.91 ± 0.03 *N* = 75	1.32 ± 0.20 *n* = 77	1.17 ± 0.12 *N* = 38	1.04 ± 0.07 *N* = 35
Mean difference	0.24 ± 0.15		0.41 ± 0.20		−0.12 ± 0.14	
95% CI	−0.051 to 0.53		0.005 to 0.82		−0.41 to 0.17	
Unpaired *t*-test *P*-value	0.106		^*^0.047		0.403	
**DMD**						
Mean	0.89 ± 0.06 *N* = 32	1.23 ± 0.14 *N* = 57	0.92 ± 0.06 *N* = 22	1.33 ± 0.21 *N* = 37	1.15 ± 0.34 *N* = 11	1.09 ± 0.11 *N* = 19
Mean difference	0.33 ± 0.19		0.41 ± 0.28		−0.06 ± 0.30	
95% CI	−0.06 to 0.72		−0.14 to 0.96		−0.67 to 0.55	
Unpaired *t*-test *P*-value	0.091		0.143		0.838	
**OD**						
Mean	1.05 ± 0.05 *N* = 87	1.01 ± 0.04 *N* = 49	0.90 ± 0.03 *N* = 54	1.02 ± 0.05 *N* = 37	1.09 ± 0.08 *N* = 25	0.98 ± 0.06 *N* = 16
Mean difference	−0.04 ± 0.07		0.12 ± 0.06		−0.10 ± 0.11	
95% CI	−0.18 to 0.11		0.007 to 0.24		−0.31 to 0.12	
Unpaired *t*-test *P*-value	0.619		^*^0.038		0.367	

* *statistically significant*.

*CI, Confidence Interval*.

Dynamometer measurement had some limitations: 8 patients could not perform the isometric elbow flexion measurement as their strength was below the lower limit of detection (DMD *N* = 2, FSHD *N* = 4, LGMD *N* = 2) and 4 patients could not perform the isokinetic knee flexion/extension measurement as their strength was below the lower limit of detection (DMD *N* = 1, FSHD *N* = 3). One DMD patient could only perform the isometric elbow extension measurement. One LGMD patient did not perform the isometric left knee extension measurement due to the onset of pain. This way, due to the lower number of observations, *t*-test analysis of upper limbs did not reach statistical significance; similarly, significance is higher in OD group than in DMD group as almost all OD patients could perform every test, while 8 DMD patients did not perform at least 1 measurement. Likewise, the majority of the analysis of single quantitative muscle strength parameters did not reach statistical significance due to the low number of observations (Supplementary Tables 2, 4), except for the isokinetic knee extension of right leg in OD subjects treated with FLAVOMEGA (Supplementary Table 3).

6MWT was performed only in OD group as DMD group include all non-walking patients. We noted a high variability in 6MWD of OD group in term of total distance walked (100–700 m). Globally, mean of total distance was lower at T-24 weeks vs. T0 in OD placebo group (mean difference + SD of total meters walked T24wk-baseline −6 ± 47.43 m), while it was higher at T-24 weeks vs. T0 in FLAVOMEGA group (mean difference ± SD of total meters walked T24wk-baseline 23.25 ± 59.67). Linear regression analysis confirmed a significant increase in meters walked in OD subjects treated with FLAVOMEGA (*P* = 0.033, regression coefficient + Standard Error (ES) 78.04 ± 30.54; CI 7.61–148.46) vs. placebo group (Table 11). No patients had accidental fall during 6MWD and all patients completed the test. No patient lost ambulation during the study.

**Table 11:** Linear regression of repeated measure (MMRM) analysis of secondary endpoints: Cpk analysis of absolute variation in serum levels (T24wk-baseline/baseline), 6MWT analysis of ΔT24wk-baseline meters walked, and performance scale analysis of ΔT24wk-baseline score.

	**DMD**		**OD**	
**CpK (U/L)**	**Placebo**	**FLAVOMEGA**	**Placebo**	**FLAVOMEGA**
Patients (*N*=)	5	7	9	6
Mean baseline	1051.00 ± 693.17	2402.29 ± 1146.79	627.00 ± 185.03	639.36 ± 642.84
Mean T 24wk (*N*=)	1199.40 ± 500.82 (5)	1320.71 ± 711.07 (7)	423.71 ± 206.05 (7)	397.97 ± 214.12 (5)
coefficient + SEM	−0.634	0.161	−0.022	0.289
*P*-value 95% CI	0.003^*^	−0.99 ± -0.270	0.94	−0.665 ± 0.621
**6MWT (m)**				
Patients (*N*=)	Np	Np	7	4
Mean baseline	Np	Np	313.57 ± 214.19	568.25 ± 130.76
Mean T 24 wk (*N*)	Np	Np	306.71 ± 180.96	591.5 ± 102.18
coefficient + SEM	Np	Np	78.04	30.54
*P*-value 95% CI	Np	Np	0.033^*^	7.61 ± 148.47
**ACTIVLIM**				
Patients (*N*=)	5	7	7	5
Mean baseline	6.2 ± 9.60	2.85 ± 4.33	24.71 ± 7.52	28.2 ± 10.82
Mean T 24 wk (*N*)	2.6 ± 3.71	3.28 ± 4.85	23.82 ± 7.11	26.8 ± 13.55
coefficient + SEM	2.54	1.41	−1	4.95E-16
*P*-value 95% CI	0.105	−0.655 ± 5.72	9.06E-135	−1 ±−1
**ABILHAND**				
Patients (*N*=)	5	7	7	5
Mean baseline	13.8 ± 12.27	15 ± 13.03	33.71 ± 2.36	28.6 ± 11.12
Mean T 24 wk (*N*)	9.8 ± 9.09	14 ± 11.07	34 ± 1.63	30.6 ± 7.46
coefficient + SEM	3.51	4.47	−0.51	1.75
*P*-value 95% CI	0.453	−6.600 ± 13.61	0.779	−4.452 ± 3.44
**EK**				
Patients (*N*=)	5	7	7	5
Mean baseline	16.6 ± 6.8	17 ± 3.91	28 ± 2.08	27.2 ± 4.65
Mean T 24 wk (*N*)	18 ± 7.31	15.85 ± 5.89	28.42 ± 1.39	28.2 ± 3.03
coefficient + SEM	−2.553	2.243	0.281	0.330
*P*-value 95% CI	0.285	−7.62 ± 2.52	0.417	−0.465 ± 1.027

* *statistically significant*.

*CI, Confidence Interval. coefficient + SEM, Regression coefficient + Standard Error of the Mean*.

**Figure 2:**
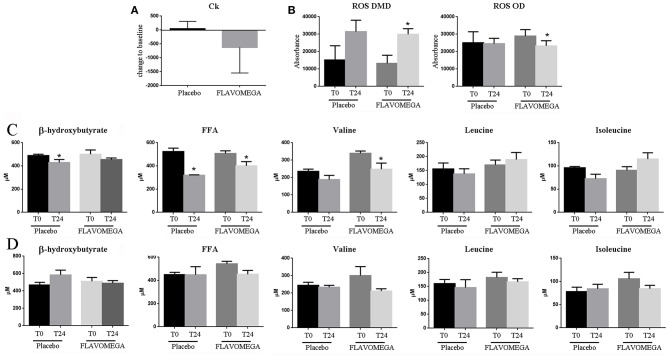
**(A)** Ck serum levels in placebo and FLAVOMEGA-treated group as ΔT24wk-T baseline. **(B)** ΔT24wk-T baseline of serum levels of ROS in treated and untreated DMD and OD groups. **(C)** ΔT24wk-T baseline of serum levels β-hydroxybutyrate, FFA, and BCAA in DMD **(C)** and OD **(D)** groups. Paired *t*-test: ^*^P < 0.05.

At week 24, we observed a decrease in serum CK concentrations for all patients treated with FLAVOMEGA vs. placebo (mean treatment differences of −670.7 ± 276.3 IU/L; 95% CI −1244 to −97.61; *P* = 0.0239). Results were confirmed by linear regression analysis and were statistically significant in DMD treated vs. DMD placebo group (Table 11 and Figure 2). Conversely, no statistically significant differences were observed in OD subjects treated with FLAVOMEGA vs. placebo. Taking in consideration the significant decrease of CK in non-ambulant DMD, we compared CK values of non-ambulant OD patients and found no differences between FLAVOMEGA and placebo group (coefficient + SEM: −0.035 ± 0.412; *P*-value 95% CI: 0.95; −0.735 ± 0.714). No gender-related differences in dynamometer, 6MWT, and CK values were found in LGMD patients.

Results from the parent questionnaire data (EK, ACTIVLIM, and ABILHAND) showed no statistical differences between FLAVOMEGA and placebo groups. DMD group showed very low score in ACTIVLIM scale as expected for age and disease severity (Table 11).

Finally, we analyzed the serum of treated and untreated patients to investigate whether the FLAVOMEGA supplementation could modulate the amount of inflammatory participants such as ROS and other markers associated to metabolites such as FFA and BCAA. We found decreased serum levels of ROS in OD subjects treated with FLAVOMEGA vs. placebo group (*P* = 0.0295) (Figure 2B). Moreover, serum levels of ROS increased in DMD treated and placebo groups, suggesting that oxidative stress was not attenuated by FLAVOMEGA treatment of DMD subjects (Figure 2B). Conversely, we found a significantly decreased of circulating levels of valine (*P* = 0.0463) and FAA (*P* = 0.0246) in DMD treated group (Figure 2C) whereas no differences were found for these biomarkers between OD treated and placebo group (Figure 2C). Studies of mitochondrial metabolism in dystrophic tissue have uncovered significant metabolic perturbations including altered substrate utilization shifting from long chain fatty acids to carbohydrates and decreased activity of various enzymes. The greater difference in valine and FAA in DMD treated group may reflect increase in energy metabolism.

## Discussion

MDs from diverse mutations such as DMD, LGMD and FSHD can produce an immune response that can amply muscle pathology (53). Advancing our understanding of the interactions between the immune system and dystrophic muscle has already led to identifying new potential immunosuppressant therapeutic strategies which will may not cure any of the muscular dystrophies but rather attenuate the severity of the disease (7, 53–58). Since nutrition is one of the major exogenous factors modulating different aspects of immune function, we recently tested the effects of natural polyphenols in dystrophin deficient animal model and demonstrated the amelioration of endurance and muscular inflammation and fibrosis (36). Following these promising evidences, we evaluated the safety and efficacy of a mix of natural polyphenols (FLAVOMEGA) in DMD, LGMD, and FSHD patients who were characterized for their muscle inflammatory background. Our data confirmed the safety of the FLAVOMEGA treatment since no withdrawals were recorded from the study because of adverse events. However, an imbalance in baseline disease severity characteristics was observed between the DMD and OD (LGMD and FSHD) arms with inability of DMD to walk and greater mean age observed in OD group. Moreover, a relatively wide age range with significant variation in muscle performance was observed in OD patients between FLAVOMEGA and placebo groups (Table 4).

These imbalances reflected the inclusion of an older and more impaired population of DMD and OD patients at baseline. However, T24 weeks analysis (paired values and MMRM) were compared to the baseline (ΔT24) reducing the bias of pre-existing difference. Interestingly, global dynamometer strength measurement was statistically increased in all FLAVOMEGA treated patients and in FLAVOMEGA treated DMD vs. placebo groups.

6MWD test of OD patients demonstrated no baseline difference between FLAVOMEGA and placebo groups (Table 4). Importantly, linear regression analysis of 6MWD changes from baseline to T24 demonstrated that the FLAVOMEGA treatment significantly improved the number of meter walked in OD patients (Table 11).

Additionally, significant improvement on the single dynamometer strength measurements was observed in FLAVOMEGA treated OD patients (Tables 6, 10 and Supplementary Tables 2, 3). Although the significant benefits in FLAVOMEGA treated OD group might be partially related to the lower mean baseline of 6MWD values of the placebo OD group, these data are relevant if we consider the expectations for a trial of 24 weeks duration performed in LGMD and FSHD patients with a long-term outcome. Moreover, early stage of disease and long-term analysis may be more appropriate to measure therapeutic effect of FLAVOMEGA in LGMD and FSHD patients.

Serum CK concentration was statistically significant reduced in DMD patients treated with FLAVOMEGA vs. placebo. Although in DMD patients CK is known to decrease with age and stage of the disease, the observed reduction was irrespective of baseline age and suggests a beneficial role in reducing muscle necrosis as previously described (59, 60). No statistically significant differences were observed for the CK values in FLAVOMEGA treated OD and placebo groups suggesting that it can be difficult to observe significant CK modification after a period of 24 weeks in ambulatory LGMD and FSHD subjects. There were no clinically relevant differences between treatment groups in clinical chemistry, hematology, or coagulation values. Dysregulations of BCAA and FAA serum levels were recently considered as biomarker of muscle disease in MD subjects (25). Interestingly, we observed the statistically significant decrease of serum levels of valine and FAA in DMD group treated with FLAVOMEGA compared to placebo (Figure 2). These data may suggest an energetic and metabolic effect of FLAVOMEGA treatment in DMD subjects. In contrast, serum levels of ROS was similar in OD groups whereas increased ROS levels were found in FLAVOMEGA and placebo treated DMD patients suggesting that FLAVOMEGA has not impact on the oxidative pathways of these patients. Although it seems that CK, BCAA, and FAA serve as plausible pharmacodynamic biomarkers for FLAVOMEGA, these data needed validation in a larger sample sizes of patients with muscular dystrophy. No statistically significant differences were observed for the EK, ACTIVLIM, and ABILHAND scales in all tested groups. The correlation between these scales and disease progression in MDs is uncertain, so these measures might not be expected to show any evidence of consistent changes across the study. The study was exploratory and was not designed to have sufficient power for any endpoint. We screened a small number of patients for each category and, therefore, variability in clinical efficacy endpoints resulted to be quite elevated. Randomization did not take into account ambulant or non-ambulant patients, or baseline 6MWD values, which could have affected functional changes. There were differences in age distribution between treated and placebo groups in DMD patients. We bear no knowledge about other randomized, placebo-controlled clinical trials testing flavonoids/omega3 supplementation in DMD, LGMD, and FSHD. A review from the literature retrieved one study of Pentoxyfillin in DMD patients that failed to demonstrate strength improvement (61). In contrast, CoQ_10_ analogs supplementation demonstrated efficacy in phase I/II (62) and in large phase III studies (63) in improving respiratory function in DMD subjects. Finally, vitamin C, E, zinc-gluconate and selenomethionine supplementation showed only partial benefit in FSHD patients improving maximum voluntary contraction of quadriceps without affecting 6MWT performance (64). A summary of nutritional intervention in DMD and mdx mice by Radley et al., highlighted the potential of amino acid and protein supplementation in improving muscle mass and the benefit associated to the use of alternative medicine such as green-tea extract (65). Our findings suggest that flavonoids/omega3 supplementation might provide clinical benefits of patients affected by MDs. Additional trials with large cohort of MDs patients will offer the opportunity to assess conclusively the efficacy of flavonoids/omega3 supplementation. Despite the acknowledged limitations, this study represents a landmark, being the first controlled diet supplementation study so far to collect muscle data with 6MWD and dynamometer tests. Diet supplementation represents a simple and available treatment, which can be used in combination with other drugs and may contribute in the maintenance of muscle function, which is regarded by dystrophic patients and their families as a meaningful outcome.

## Data Availability

The raw data supporting the conclusions of this manuscript will be made available by the authors, without undue reservation, to any qualified researcher.

## Ethics Statement

This phase I/II, randomized double-blind placebo-controlled clinical study was conducted at the Fondazione IRCCS Ca' Granda Ospedale Maggiore Policlinico, Milan, Italy. Each patient (or patient's parents) gave the written informed consent for the research. This study was performed in accordance with International Conference on Harmonisation of Good Clinical Practice guidelines, the Declaration of Helsinki (2008) and the European Directive 2001/20/EC. This monocenter study was approved by the Ethical Committee at Fondazione IRCCS Ca' Granda Ospedale Maggiore Policlinico of Milan, under the acronym PRO1. This trial was registered on ClinicalTrials.gov with the following number: NCT03317171.

## Author Contributions

YT and CS conceived and designed the experiments. AF, YT, and CS wrote the paper. PB and CV interpreted and analyzed the data. MB, MM, MA, and DB performed the experiments and acquired the data. LV performed statistical analysis. All the authors stated were involved in the critical revision of the manuscript and approved the final version of the article, including the authorship list. The corresponding author had full access to all the data in the study and had final responsibility for the decision to submit for publication.

### Conflict of Interest Statement

The authors declare that the research was conducted in the absence of any commercial or financial relationships that could be construed as a potential conflict of interest.
