# Supplementary material for: Preliminary Evidences of Safety and Efficacy of Flavonoids- and Omega 3-Based Compound for Muscular Dystrophies Treatment: A Randomized Double-Blind Placebo Controlled Pilot Clinical Trial
Source: Front Neurol. 2019 Jul 23;10:755. doi: 10.3389/fneur.2019.00755 (PMC6664031; doi:10.3389/fneur.2019.00755)
Supplement: Supplementary file 1 [file Data_Sheet_1.docx]

**Supporting information**

**Supplementary Figures Legend**

**Supplementary Figure 1** Quantitative dynamometer strength analysis of all muscular districts paired baseline vs T24 weeks in all patients or DMD or OD groups pre- and post- placebo/FLAVOMEGA.

Paired t-test: ***P<0,001

**Supplementary Figure 2** Quantitative dynamometer strength analysis of lower and upper arms related to baseline in all patients or DMD or OD groups pre- and post- placebo/FLAVOMEGA.

Student t-test: *P<0,05

**Supplementary Tables Legend**

**Supplementary Table 1** Patients’ strength measurement at baseline after stratification

|  | **DMD** | | | | | |  | **OD** | | | | | | |
| --- | --- | --- | --- | --- | --- | --- | --- | --- | --- | --- | --- | --- | --- | --- |
|  | **Placebo** | | | **FLAVOMEGA** | | |  | **Placebo** | | | **FLAVOMEGA** | | |  |
| **T24wk Per Protocol analysis** | **Mean** | **SD** | **N** | **Mean** | **SD** | **N** | **p value** | **Mean** | **SD** | **N** | **Mean** | **SD** | **N** | **p value** |
| Global strength | **4,34** | **2,24** | **5** | **5,26** | **3,69** | **7** | **0,42** | **19,40** | **17,89** | **7** | **81,13** | **62,78** | **5** | **0,21** |
| Isometric knee extension R | 6,17 | 1,52 | 4 | 5,54 | 4,65 | 7 | 0,80 | 29,03 | 29,16 | 7 | 182,26 | 124,55 | 5 | *0,009 |
| Isometric knee extension L | 5,42 | 2,09 | 4 | 4,81 | 3,14 | 7 | 0,90 | 35,00 | 33,49 | 7 | 135,08 | 115,36 | 5 | 0,05 |
| Isometric knee flexion R | 8,44 | 6,96 | 3 | 6,66 | 8,62 | 7 | 0,76 | 10,66 | 11,68 | 7 | 69,75 | 55,35 | 5 | *0,019 |
| Isometric knee flexion L | 6,95 | 2,42 | 2 | 8,71 | 10,71 | 5 | 0,84 | 11,65 | 12,58 | 7 | 75,95 | 49,33 | 4 | *0,008 |
| Isokinetic knee extension R | 6,95 | 1,23 | 3 | 8,38 | 2,40 | 3 | 0,41 | 25,17 | 27,16 | 7 | 151,93 | 121,08 | 5 | *0,021 |
| Isokinetic knee flexion R | 8,75 | 3,49 | 3 | 13,76 | 8,83 | 3 | 0,41 | 13,01 | 10,06 | 7 | 73,76 | 52,57 | 5 | *0,012 |
| Isokinetic knee extension L | 5,30 | 0,94 | 3 | 8,69 | 2,49 | 3 | *0,092 | 32,06 | 31,97 | 7 | 110,66 | 102,79 | 5 | *0,082 |
| Isokinetic knee flexion L | 6,02 | 0,51 | 3 | 9,00 | 4,54 | 3 | 0,32 | 18,61 | 17,86 | 7 | 57,26 | 47,56 | 5 | *0,074 |
| Isometric elbow extension R | 2,00 | 1,31 | 5 | 3,90 | 2,26 | 7 | 0,17 | 13,87 | 11,15 | 7 | 26,90 | 27,26 | 5 | 0,28 |
| Isometric elbow extension L | 2,93 | 1,10 | 5 | 3,52 | 2,80 | 7 | 0,67 | 14,36 | 11,70 | 7 | 25,82 | 27,63 | 5 | 0,34 |
| Isometric elbow flexion R | 2,40 |  | 1 | 5,17 | 0,57 | 3 |  | 15,62 | 17,04 | 7 | 62,59 | 25,22 | 4 | *0,004 |
| Isometric elbow flexion L | 2,95 |  | 1 | 3,97 | 1,50 | 3 |  | 13,73 | 14,22 | 7 | 32,78 | 25,87 | 4 | 0,14 |

P value is intended as unpaired t-test P value

* statistically significant

**Supplementary Table 2** Biodex measurements of isometric knee extension/flexion right/left of Placebo and FLAVOMEGA treated groups.

| Strength (T24wk/baseline) | **Isometric knee extension right** | | **Isometric knee extension left** | | **Isometric knee flexion right** | | **Isometric knee flexion left** | |
| --- | --- | --- | --- | --- | --- | --- | --- | --- |
| **All Patient** | **Placebo** | **FLAVOMEGA** | **Placebo** | **FLAVOMEGA** | **Placebo** | **FLAVOMEGA** | **Placebo** | **FLAVOMEGA** |
| Mean | 1,01±0,04 N=8 | 1,81±0,54 N=11 | 0,78±0,067 N=10 | 0,90±0,11 N=11 | 0,75±0,068 N=10 | 0,78±0,14 N=11 | 0,945±0,12 N=10 | 0,87±0,15 N=8 |
| MD | 0,81±0,64 | | 0,12±0,14 | | 0,028±0,16 | | -0,08±0,18 | |
| 95% CI | -0,54 to 2,15 | | -0,31 to 0,36 | |  |  | -0,47 to 0,31 | |
| P value |  | 0,22 | 0,39 | | 0,86 | | 0,67 | |
| **DMD** | **Placebo** | **FLAVOMEGA** | **Placebo** | **FLAVOMEGA** | **Placebo** | **FLAVOMEGA** | **Placebo** | **FLAVOMEGA** |
| Mean | 1,02±0,07 N=4 | 4,19±1,86 N=7 | 0,52±0,11 N=3 | 0,92±0,20 N=6 | 0,85±0,16 N=3 | 0,53±0,14 N=6 | 1,07±0,21 N=2 | 0,81±0,31 N=4 |
| MD | 3,17±2,52 | | 0,40±0,31 | | -0,32±0,23 | | -0,26±0,48 | |
| 95% CI | -2,52 to 8,86 | | -0,33 to 1,12 | | -0,86 to 0,22 | | -1,58 to 1,07 | |
| P value | 0,24 | | 0,23 | | 0,20 | | 0,62 | |
| **OD** | **Placebo** | **FLAVOMEGA** | **Placebo** | **FLAVOMEGA** | **Placebo** | **FLAVOMEGA** | **Placebo** | **FLAVOMEGA** |
| Mean | 0,99±0,04 N=4 | 0,98±0,08 N=5 | 0,89±0,03 N=7 | 0,88±0,09 N=5 | 0,71±0,07 N=7 | 1,07±0,20 N=5 | 0,92±0,14 N=8 | 0,93±0,05 N=4 |
| MD | -0,01±0,09 | | -0,013±0,09 | | 0,37±0,19 | | 0,01±0,2 | |
| 95% CI | -0,23 to 0,21 | | -0,21 to 0,19 | | -0,05 to 0,78 | | -0,44 to 0,46 | |
| P value | 0,92 | | 0,89 | | 0,08 | | 0,96 | |

P value is intended as unpaired t-test P value

**Abbreviations**:

**CI**: Confidence Interval

**MD**: mean difference

**Supplementary Table 3** Biodex measurements of isokinetic knee extension/flexion right/left of Placebo and FLAVOMEGA treated groups.

| Strength (T24wk/baseline) | **Isokinetic knee extension right** | | | **Isokinetic knee extension left** | | **Isokinetic knee flexion right** | | | **Isokinetic knee flexion left** | |
| --- | --- | --- | --- | --- | --- | --- | --- | --- | --- | --- |
| **All Patient** | **Placebo** | | **FLAVOMEGA** | **Placebo** | **FLAVOMEGA** | **Placebo** | | **FLAVOMEGA** | **Placebo** | **FLAVOMEGA** |
| Mean | 0,92±0,05 N=11 | | 1,25±0,23 N=8 | 1,04±0,09 N=9 | 1,28±0,41 N=9 | 0,81±0,06 N=10 | | 1,19±0,19 N=8 | 1,03±0,08 N=9 | 1,22±0,20 N=9 |
| MD | 0,33±0,2 | | | 0,24±0,42 | | 0,38±0,18 | | | 0,19±0,22 | |
| 95% CI | -0,10 to 0,77 | | | -0,65 to 1,12 | | -0,003 to 0,78 | | | -0,27 to 0,65 | |
| P value | 0,12 | | | 0,58 | | 0,05* | | | 0,40 | |
| **DMD** | **Placebo** | **FLAVOMEGA** | | **Placebo** | **FLAVOMEGA** | **Placebo** | **FLAVOMEGA** | | **Placebo** | **FLAVOMEGA** |
| Mean | 0,92±0,13 N=4 | 1,32±0,47 N=4 | | 1,11±0,10 N=2 | 1,67±0,90 N=4 | 0,91±0,17 N=3 | 1,43±0,33 N=4 | | 1,06±0,01 N=2 | 1,23±0,47 N=4 |
| MD | 0,4±0,49 | | | 0,56±1,36 | | 0,49±0,42 | | | 0,17±0,70 | |
| 95% CI | -0,80 to 1,61 | | | -3,21 to 4,3 | | -0,57 to 1,57 | | | -1,77 to 2,11 | |
| P value | 0,44 | | | 0,70 | | 0,28 | | | 0,82 | |
| **OD** | **Placebo** | **FLAVOMEGA** | | **Placebo** | **FLAVOMEGA** | **Placebo** | **FLAVOMEGA** | | **Placebo** | **FLAVOMEGA** |
| Mean | 0,89±0,04 N=8 | 1,18±0,16 N=4 | | 1,02±0,11 N=7 | 0,96±0,23 N=5 | 0,76±0,06 N=7 | 0,97±0,16 N=4 | | 1,02±0,11 N=7 | 1,20±0,13 N=5 |
| MD | 0,29±0,12 | | | -0,06±0,23 | | 0,21±0,14 | | | 0,19±0,17 | |
| 95% CI | 0,02 to 0,56 | | | -0,57 to 0,46 | | -0,11 to 0,52 | | | -0,19 to 0,56 | |
| P value | 0,039* | | | 0,82 | | 0,17 | | | 0,29 | |

* statistically significant

P value is intended as Unpaired T Test- P value

**Abbreviations**:

**CI**: Confidence Interval

**MD**: mean difference

**Supplementary Table 4** Biodex measurements of isometric elbow extension/flexion right/left of Placebo and FLAVOMEGA treated groups.

| Strength (T24wk/baseline) | **Isometric elbow extension right** | | | **Isometric elbow extension left** | | **Isometric elbow flexion right** | | **Isometric elbow flexion left** | |
| --- | --- | --- | --- | --- | --- | --- | --- | --- | --- |
| **All Patient** | **Placebo** | | **FLAVOMEGA** | **Placebo** | **FLAVOMEGA** | **Placebo** | **FLAVOMEGA** | **Placebo** | **FLAVOMEGA** |
| Mean | 0,97±0,14 N=10 | | 1,04±0,10 N=12 | 1,29±0,29 N=12 | 1,05±0,14 N=12 | 0,81±0,09 N=7 | 1,01±0,10 N=5 | 1,22±0,16 N=9 | 1,06±0,19 N=6 |
| MD | 0,071±0,16 | | | -0,24±0,32 | | 0,19±0,14 | | -0,16±0,25 | |
| 95% CI | -0,27 to 0,41 | | | -0,91 to 0,43 | | -0,12 to 0,50 | | -0,70 to 0,38 | |
| P value | 0,67 | | | 0,47 | | 0,19 | | 0,53 | |
| **DMD** | **Placebo** | | **FLAVOMEGA** | **Placebo** | **FLAVOMEGA** | **Placebo** | **FLAVOMEGA** | **Placebo** | **FLAVOMEGA** |
| Mean | 0,99±0,33 N=4 | | 1,06±0,16 N=7 | 1,54±0,71 N=5 | 1,04±0,23 N=7 | 0,46 N=1 | 1,02 N=3 | 0,57 N=1 | 1,46 N=3 |
| MD | 0,07±0,32 | | | -0,49±0,64 | | /^§^ | | /^§^ | |
| 95% CI | -0,66 to 0,80 | | | -1,9 to 0,94 | | /^§^ | | /^§^ | |
| P value | 0,83 | | | 0,46 | | /^§^ | | /^§^ | |
| **OD** | **Placebo** | **FLAVOMEGA** | | **Placebo** | **FLAVOMEGA** | **Placebo** | **FLAVOMEGA** | **Placebo** | **FLAVOMEGA** |
| Mean | 0,96±0,11 N=6 | 1,02±0,11 N=5 | | 1,11±0,13 N=7 | 1,06±0,16 N=5 | 0,87±0,08 N=6 | 0,99±0,10 N=2 | 1,30±0,15 N=8 | 0,86±0,05 N=4 |
| MD | 0,06±0,15 | | | -0,05±0,20 | | 0,12±0,16 | | -0,44±0,23 | |
| 95% CI | -0,29 to 0,40 | | | -0,51 to 0,41 | | -0,27 to 0,50 | | -0,95 to 0,06 | |
| P value | 0,27 | | | 0,82 | | 0,49 | | 0,08 | |

P value is intended as Unpaired T Test- P value

^§^values are incalculable due to paucity of observations

**Abbreviations**:

**CI**: Confidence Interval

**MD**: mean difference
