# Supplementary figures and images for: Preliminary Evidences of Safety and Efficacy of Flavonoids- and Omega 3-Based Compound for Muscular Dystrophies Treatment: A Randomized Double-Blind Placebo Controlled Pilot Clinical Trial
Source: Front Neurol. 2019 Jul 23;10:755. doi: 10.3389/fneur.2019.00755 (PMC6664031; doi:10.3389/fneur.2019.00755)

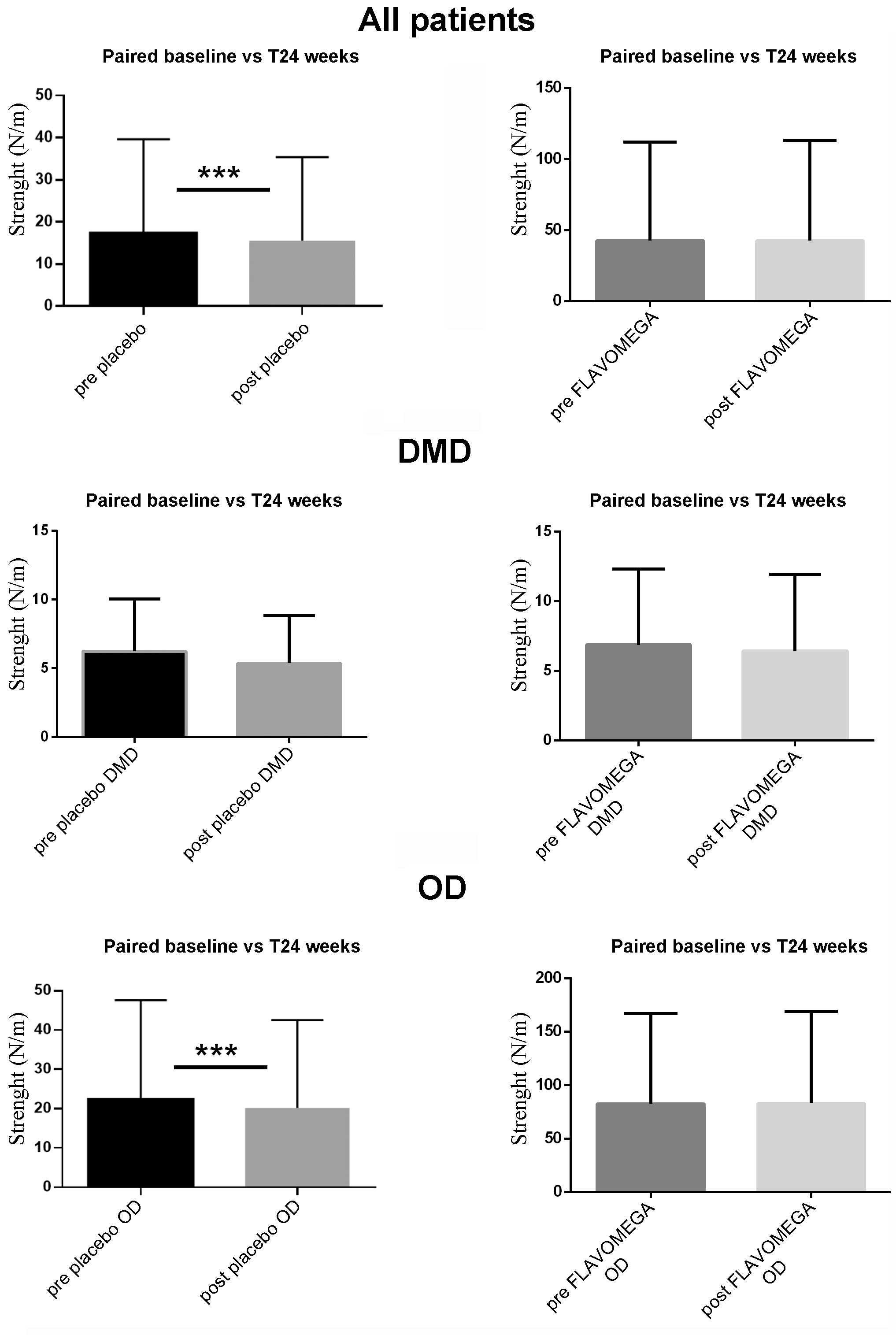

Supplement: Supplementary file 2 [file Image_1.JPEG]

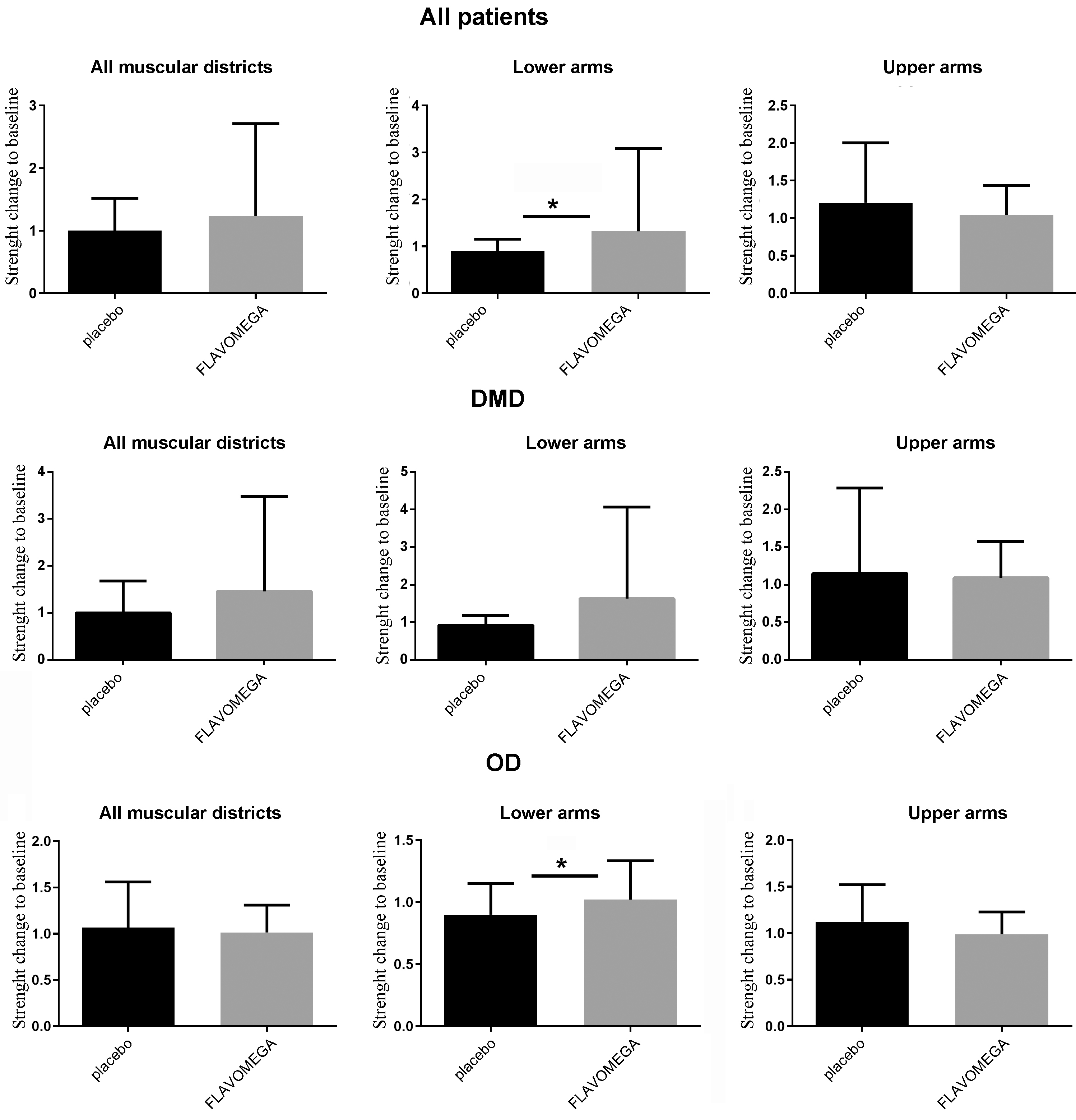

Supplement: Supplementary file 3 [file Image_2.JPEG]
